# The Colour Problem in Hospital Life

**Published:** 1914-07-11

**Authors:** 


*To the Editor of* The Hospital.

### The Colour Problem in Hospital Life.

*To the Editor of* The Hospital.

Sir,—The valuable article upon this topic which an "Officer of the London Hospital" contributes to your pages raises a very old subject of discussion amongst students, though it is usually debated in private rather than in public. The views adopted by your contributor from the East End may possibly not find favour with the majority of present-day students, as indeed they would have been rejected by me in days of pupilage. Since then, however, I have wandered in many quarters of the globe, and met many coloured races, Asiatic, African, and American; and my views are closely in accord with those to which you have afforded publicity. At present the question affects chiefly the educated Hindu, Malayan, Chinese, and Japanese students. These I would have no hesitation in admitting to our medical schools on Imperial grounds, even if on no other. They are usually intelligent and industrious. Medical training they must have, somehow or other; and if we refuse it them they will get it elsewhere. They may, in such an event, imbibe ideas which are hostile to our race; and in any case they will fail to derive that element of British education, the sense of fair play and sportsmanship, which is so difficult to acquire on the Continent. If any example is needed, one can point to Dr. G. L. Tuck (Wu Lien Teh), a most distinguished St. Mary's Hospital man, whose popularity as a student among his English colleagues was as pronounced as his after-record in China has been brilliant.

There remains the negro. What the future has in store for this race, or conglomeration of races, he would be over-bold who would foretell. In spite of a sincere affection for a few of the African tribes who are as yet but little contaminated by contact with civilisation, my experience compels me to look with grave misgivings upon the attendance of white women by negroes. Here, for the present, I would draw the line, in spite of the appearance of illogicality which such a course entails. But knowing what I know of negro mentality I cannot contemplate without misgiving for men of that race the privileges I would grant to other coloured peoples. I may be inconsistent and prejudiced, but I fancy the Hindu and the Chinese students would take the same view as does yours faithfully, Capricorn.

